# Optimization of ultrasonic‐assisted extraction of soluble dietary fiber (β‐glucan) from different barley varieties and study of its characterization and functional attributes

**DOI:** 10.1002/fsn3.4421

**Published:** 2024-09-04

**Authors:** Nomeena Anis, Zaheer Ahmed, Nauman Khalid

**Affiliations:** ^1^ Department of Nutritional Sciences & Environmental Design Allama Iqbal Open University (AIOU) Islamabad Pakistan; ^2^ Department of Food Science and Technology, School of Food and Agricultural Sciences University of Management and Technology Lahore Pakistan; ^3^ College of Health Sciences Abu Dhabi University Abu Dhabi United Arab Emirates

**Keywords:** green extraction, polysaccharides, RSM, sonication

## Abstract

Green technology, encompassing sustainable practices in food production, extends to dietary fiber extraction. This study aimed to enhance dietary fiber extraction from the selected barley varieties (Jou‐17, Sultan‐17, and Pearl‐21) using the ultrasonic‐assisted extraction (UAE) technique. This process involved washing, drying, de‐fatting (using ethanol as green solvent), and protein removal steps. The response surface methodology (RSM) technique was used to optimize the yield of soluble dietary fiber (SDF; β‐glucan) with time, temperature, and power. Optimal conditions yielded the highest SDF (5.21%) in all selected varieties after 17.5 min at 41.70°C with 130.5 W. FTIR pattern confirmed the functional group in the tested sample. TGA and DSC spectra determined the thermal of SDF (β‐glucan). Monosaccharide composition confirmed that SDF (β‐glucan) is glucose in nature. Proximate analysis indicated that Jou‐17 had the highest moisture (13.4%) and crude fiber (10.10%) content. Sultan‐17, on the other hand, had the maximum levels of ash (2.75%), crude fat (1.22%), and protein (8.84%). The NFE, water‐holding capacity, oil‐holding capacity, and foaming capacity of extracted SDF (β‐glucan) in the “Pearl‐21” barley variety were determined to be 78.37%, 14.07 g/g, 6.99 g/g, and 126.17%, whereas highest foaming‐stability (96.26%) was observed in Jou‐17 variety. PCA also confirmed the association in studied variables. In a nutshell, optimizing the extraction of SDF (β‐glucan) from the selected barley varieties using green technology and its favorable properties opens up promising paths for future endeavors and contributes to the advancement of sustainable and health‐conscious practices in the food industry.

## Graphical abstract

This study successfully enhanced the extraction of soluble dietary fiber (SDF; β‐glucan) from specific barley varieties using ultrasonic‐assisted extraction technique. Through response surface methodology, optimal conditions were determined to yield the highest SDF content, and the extracted SDF was characterized for its properties and composition. The findings demonstrate the potential for sustainable and health‐conscious applications of this green technology in the food industry, emphasizing its contribution to environmentally friendly and nutritionally beneficial practices.

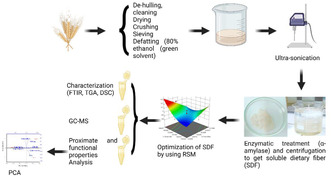

## 1 INTRODUCTION

Barley (*Hordeum vulgare* L.) is one of the most demanding cereal crops categorized within the Poaceae family (Order: Poales). Barley is predominantly employed for animal feed and brewing purposes; it also holds significance as a primary food source in regions where cultivating other major cereals is not feasible (Guo et al., 2020). The choice of barley as the experimental material was likely driven by its multifaceted attributes and potential applications (Hill et al., 2021). Barley exhibits a wide range of varieties, each with distinct attributes, allowing researchers to investigate variations in properties and functionalities across different types of barley. In addition, barley demonstrates commendable adaptability to unfavorable conditions such as cold, drought, or nutrient‐deficient soils. It is notably more resilient than wheat when facing adverse growing circumstances (Giraldo et al., 2019). In 2019, both Jou‐17 and Sultan‐17 varieties received approval from the Punjab Seed Council (PSC) for general cultivation because of their outstanding performance in higher grain yield, rust resistance, and superior nutritional quality (Riaz et al., 2022). The average yield performance of Jou‐17 and Sultan‐17 varieties was measured at 4262 and 4317 kg/ha respectively. Meanwhile, the yield of the Pearl‐21 variety was reported as 3655 kg/ha. The seeds of all selected barley varieties were planted well in rainfed (arid zone) and normal irrigated conditions (Hanif et al., 2024).

Dietary fiber is a complex carbohydrate in barley that has physiological health benefits (Li & Komarek, 2017). Soluble dietary fiber (SDF) and insoluble dietary fiber (IDF) are the two main categories of dietary fibers (Wang et al., 2021). The IDF usually has more content than the SDF in barley (Bader Ul Ain et al., 2019). However, barley is also rich in β‐glucan (SDF), a water‐soluble polysaccharide, averaging up to 6% concentration with a linear chain of β‐glucopyranosyl units comprising about 70% (1 → 4) linkages and 30% (1 → 3) linkages (Goudar et al., 2020). Recently, SDF (β‐glucan) has become a more functional option than IDF because it effectively drops cholesterol, triglyceride, and glucose levels in the blood (Bader Ul Ain et al., 2020). Numerous health advantages come from increasing dietary fiber intake, including a decreased risk of diabetes, obesity, heart disease, and several cancers (Barber et al., 2020; Li & Komarek, 2017). Additionally, dietary fiber can offer functional qualities to foods such as higher water‐holding capacity (WHC) and oil absorption, emulsification, and/or gel formation (Siddiqui et al., 2023). Furthermore, dietary fiber has the potential to be a food additive or functional food ingredient to fulfill the technological requirements for producing value‐added products (Sagar et al., 2018).

The characteristics of dietary fibers are influenced by various extraction techniques. The extraction method is determined by its chemical composition, number of oligosaccharides, complexity, level of polymerization, and other factors. The extraction conditions, including the solid–liquid ratio, temperature, and contact time, have an impact on the SDF (β‐glucan) yield and properties, which affect how it can be used in food and its impact on the human body (Hussain et al., 2020). The use of green technologies protects the environment and natural resources. In recent years, environment‐friendly extraction techniques have become popular, including extraction by water, extraction by steam, ultrasonic‐assisted extraction, ethanol extraction, and their combinations (Maphosa & Jideani, 2016; Soquetta et al., 2018). Ultra‐sonication has several benefits for extraction processes that are involved in extracting various natural compounds (Rodríguez‐Pérez et al., 2015). High‐intensity ultrasonic treatments often depolymerize the extracted polymer and low molecular weight (MW) solutions which are homogenous (Mohan et al., 2022). Different MW polysaccharides' structures have been dissolved by sonication, and it was used in the regulation of the starch solution's viscosity (Xu et al., 2022).

The goal of the current research was to improve the extraction of SDF from barley cultivars (Jou‐17, Sultan‐17, and Pearl‐21) using an ultrasonic‐assisted enzymatic extraction technique. It was proposed that an optimal combination of factors be used to maximize the yield of extracted fiber by using the response surface methodology (RSM). Furthermore, the extracted SDF (β‐glucan) was characterized based on its spectroscopic, proximate, and functional properties. The PCA technique was employed to assess the association among studied variables in terms of proximate and functional properties.

## 2 MATERIALS AND METHODS

### 2.1 Procurement and preparation of sample

Barley grains (Jou‐17, Sultan‐17, and Pearl‐21) were procured from the Ayub Agriculture Research Institute, Faisalabad, Pakistan. The barley samples underwent a series of preparatory steps: dehulling, cleaning, drying, crushing, and sieving through a 10‐mesh sieve. Subsequently, the samples were defatted using 80% ethanol (green solvent) at a ratio of 1:5 w/v at room temperature for 12 h. The ethanol used before the sonication process made the dietary fiber separation much easier (El Halal et al., 2019). After discarding the solvent and effectively draining the fats, the remaining sample was transferred to another beaker and immersed in 1 L of distilled water.

### 2.2 Ultrasonic‐assisted enzymatic method to extract soluble dietary fiber

**FIGURE 1:**
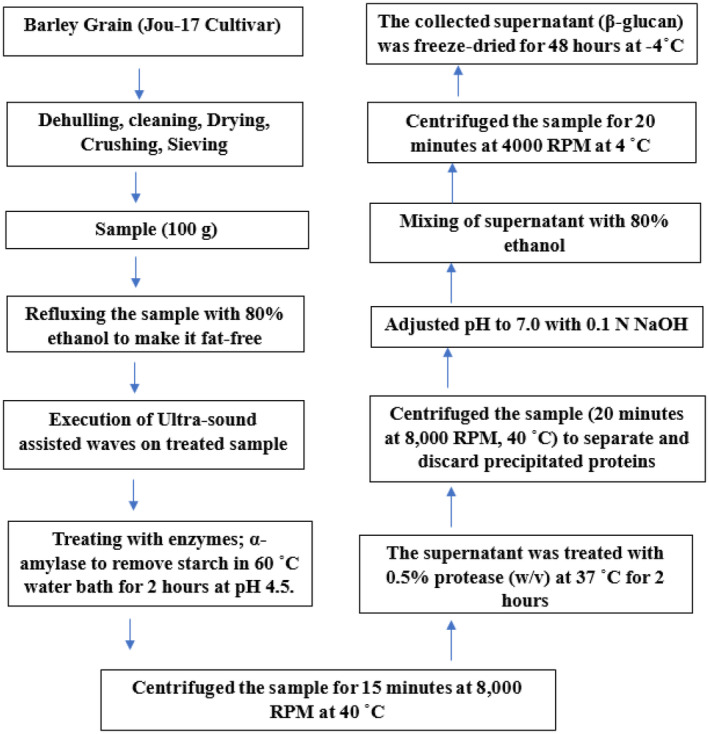
A schematic depiction of the procedure to extract SDF (β‐glucan) from selected barley varieties (Jou‐17, Sultan‐17, and Pearl‐21).

The fat‐free sample was then subjected to 20 min of ultrasound‐assisted extraction using Cole Parmer's (USA) Sonicator (Power 220‐230 VAC 50/60 HZ Cat No. C2‐04711‐45S/N. 535529 Y). A further purification step was employed to eliminate starch residue, utilizing 1% α‐amylase (w/v) in a 60°C water bath for 2 h at pH 4.5. The mixture was subsequently centrifuged for 15 min at 8000 RPM at 40°C. The supernatant underwent treatment with 0.5% protease (w/v) at 37°C for 2 h and was then centrifuged again (20 min at 8000 RPM, 40°C) to separate and discard precipitated proteins. The pH was adjusted to 7.0 by adding 0.1 N NaOH dropwise. The supernatant was further mixed with 80% ethanol for 20 min to yield SDF (β‐glucan). The sample was further centrifuged at 4000 RPM for 20 min at 4°C. The supernatant was collected and freeze‐dried (vertical Freeze Dryer BK‐FD12P, China) for 48 h at −4°C. The final product was stored for further analysis. A schematic depiction of the procedure to extract SDF (β‐glucan) has been presented in Figure 1.

### 2.3 Optimization of SDF (β‐glucan) yield

A three‐factor‐five‐level central composite design (CCD) was employed to optimize the yield of ultrasonicated SDF (β‐glucan) from selected barley varieties (Jou‐17, Sultan‐17, and Pearl‐21). The three independent variables were extraction temperature (°C), extraction time (min), and power (W). A total of 19 experiments (runs) were designed based on the CCD to optimize the yield of β‐glucan as shown in Table 1. Each experiment was conducted in triplicate, and the average yield of SDF (β‐glucan) (%) was taken as the response, denoted as Y. Regression analysis was performed on the experimental data, and it was fitted into an empirical second‐order polynomial model for further analysis.

(1) $$\begin{array}{c} \text{Yield}\;\left(\%\right)\;=\beta_{0}+\beta_{1}X_{1}+\beta_{2}X_{2}+\beta_{3}X_{3}+\beta_{12}X_{12}+\beta_{13}X_{13}+\\ \beta_{23}X_{23}+\beta_{11}X_{11}+\beta_{22}X_{22}+\beta_{33}X_{33} \end{array}$$

**TABLE 1:** Central composite design, coded levels, and observed responses.

	+α	+1	0	−1	−α
Part A (coded levels)					
A: Time	21.70	20	17.5	15	13.29
B: Temperature	41.70	35	37.5	40	33.29
C: Power	146.47	121	130.5	140	114.52

Run	Independent variable			SDF (β‐glucan) yield (%)		
	A: Time (min)	B: Temperature (°C)	C: Power (W)	Jou‐17	Sultan‐17	Pearl‐21
Part B (observed responses)						
1	15	40	121	4.91	4.99	4.95
2	17.5	37.5	146.477	5.2	5.03	5.11
3	17.5	37.5	114.523	4.92	5.01	4.99
4	17.5	41.7045	130.5	5.21	5.29	5.19
5	17.5	37.5	130.5	4.9	5.03	5.01
6	20	35	140	4.91	5.06	5.02
7	20	40	140	5.13	5.17	5.09
8	15	40	140	5.21	5.27	5.19
9	17.5	33.2955	130.5	4.99	5.08	5.03
10	17.5	37.5	130.5	4.97	5.01	5.01
11	20	40	121	5.14	5.16	5.12
12	21.7045	37.5	130.5	4.94	5.02	5.03
13	15	35	140	5.15	5.13	5.1
14	15	35	121	5.17	5.14	5.11
15	17.5	37.5	130.5	4.98	5.03	5.03
16	20	35	121	5.11	5.09	5.07
17	17.5	37.5	130.5	4.96	5.04	5
18	13.2955	37.5	130.5	4.93	5.02	4.97
19	17.5	37.5	130.5	5.04	5.11	5.05

## 3 CHARACTERIZATION OF SDF (Β‐GLUCAN)

### 3.1 Fourier‐transformed infrared spectroscopy (FTIR)

The study investigated the structural properties of SDF (β‐glucan) extracted from selected barley varieties using Fourier‐transformed infrared spectroscopy (FTIR). The SDF (β‐glucan) samples were prepared as KBr pellets at a 1:100 ratio and analyzed in the 4000–400 cm^−1^ spectral range with a 4 cm^−1^ resolution using a Bruker Vector 22 instrument, Germany. Data processing was carried out using Bruker OPUS software.

### 3.2 Sugar composition analysis

For sugar identification, SDF (β‐glucan) was subjected to hydrolysis using a 2MTFA treatment at 120°C for 2 h and then analyzed with a Varian GC/MS 4000 instrument equipped with a VF‐5 ms column as described by Afreen et al. (2023).

### 3.3 Differential scanning calorimeter (DSC)

The thermal characteristics of SDF (β‐glucan) were assessed with a differential scanning calorimeter (DSC Model 141 from SETARAM Scientific & Industrial Equipment Co Ltd., France). The dried SDF (β‐glucan) sample (4.2 g) was sealed in an aluminum pan and subjected to analysis, with an empty pan serving as the reference, to determine the melting point and enthalpy change, employing a heating rate of 10°C per minute within the temperature range of 20–300°C.

### 3.4 Thermogram analysis (TGA)

A 10 mg sample of SDF (β‐glucan) was loaded into a platinum crucible and subjected to a linear heating rate of 10°C per minute over a temperature range of 25–1000°C to investigate its thermal behavior.

### 3.5 Proximate analysis

The moisture content in the SDF (β‐glucan) of barley varieties (Jou‐17, Sultan‐17, and Pearl‐21) was assessed by adopting the AACC (2000) method no. 44‐19. The protein content was calculated by method no. 46‐10 described in AACC (2000). Kjeldhal method is generally reliable and acceptable to determine crude protein content samples. Fat contents were assessed by using the procedure as explained in AACC (2000) method no. 30‐10 by Soxhlet apparatus. Similarly, fiber contents were assessed by the procedure explained in AACC (2000) method no. 32‐10.01. Further, the ash content was determined as a total inorganic matter by the following method as depicted in AACC (2000) method no. 08‐01.

## 4 FUNCTIONAL PROPERTIES

### 4.1 Water and oil‐holding capacity

WHC and oil‐holding capacity (OHC) were determined in accordance with the methodology outlined in the research conducted by Vazquez Encalada and Segura Campos (2020), with specific modifications. One gram of the sample was introduced into a solution consisting of 20 mL of distilled water and 10 mL of corn oil, all maintained at ambient room temperature. This mixture was subjected to vigorous agitation using a vortex for 2 min. Subsequently, the sample was centrifuged at 5000 RPM for 30 min, and the volume of the resulting supernatant was measured.

### 4.2 Foaming capacity and stability

The functional characteristics of SDF (β‐glucan), were assessed by its foaming capacity and stability, in accordance with the methodology described by Temelli (1997). A 2.5‐g sample of SDF (β‐glucan) was dispersed in distilled water and subsequently subjected to whip for 2 min a Cole‐Parmer homogenizer at 10,000 rpm. The volume of the solution was recorded both before and after the mixing process.

(2) $$\text{Foaming}\;\text{capacity}\;\left(\%\right)=\frac{\left(V_{2}-V_{1}\right)}{V_{1}}\;\times \;100\%$$

To determine foam stability (FS), the foams generated were gently transferred to a 1000‐mL graduated cylinder. The volume of foam that remained after staying at 25 ± 2°C for 2 h was expressed as a percentage of the initial foam volume.

### 4.3 Statistical analysis

Using Stat‐Ease Design Expert software, version 11, the central composite design (CCD) model was undertaken to optimize the ultrasonic extraction conditions of SDF (β‐glucan) of selected barley varieties (Jou‐17, Sultan‐17, and Pearl‐21). Three independent variables (extraction temperature, time, and power) were used to run the CCD model. Quadratic equation, regression (*R*), cumulative variance (C.V), and ANOVA were calculated by Stat‐Ease Design Expert software, version 11 whereas the remaining study elements were analyzed through mean and standard deviation, with the use of statistical software (SPSS version 16.00). Furthermore, the PCA was employed using OriginLab (Ver. 2019b).

## 5 RESULTS AND DISCUSSION

### 5.1 Optimized extraction of SDF (β‐glucan)

Through the utilization of multiple regression analyses on the experimental data, the second‐order polynomial Equation (1) was derived, establishing a relationship between the response variable and the independent variables

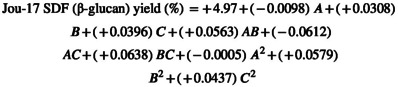




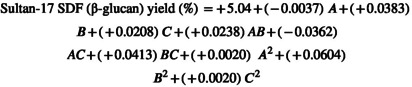




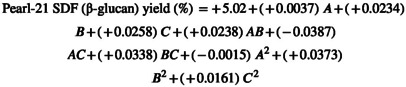




The analysis of variance (ANOVA) and fit statistics for the selected quadratic predictive model of extraction yield (*Y*) are presented in Table 2. Upon fitting the model, it was observed that the F‐value for the lack of fit proved to be nonsignificant (*p* > .05), thereby confirming the validity of the model. Additionally, the low coefficient of variation (CV) values of 1.53%, 1.12%, and 0.90% in the Jou‐17, Sultan‐17, and Pearl‐21 barley varieties clearly pointed to a very high degree of precision and a substantial level of reliability in the experimental values. The *p*‐values (Prob > *F*) were .03 and .04 affirming the significance of the model.

**TABLE 2:** ANOVA for the fitted quadratic polynomial model of extraction of SDF (β‐glucan) from selected barley varieties (Jou‐17, Sultan‐17, and Pearl‐21).

Source	Jou‐17		Sultan‐17		Pearl‐21	
	*F*‐value	*p*‐value	*F*‐value	*p*‐value	*F*‐value	*p*‐value
Model	3.53	.0372*	3.60	.0351*	3.39	.0418*
Lack of fit	3.49	.1248	3.17	.1432	8.64	.1288
*R* ^2^	0.7791		0.7824		0.7721	
C.V (%)	1.53		1.12		0.90	

* *p* < .05.

The regression coefficients for Equation (1) are detailed in the accompanying Table 3. Notably, the *p*‐value corresponded to a more pronounced significance of the respective coefficient, a principle described by Du et al. (2014).

**TABLE 3:** Regression model analysis of SDF (β‐glucan) yield in relation to independent variables.

Source	Jou‐17		Sultan‐17		Pearl‐21	
	*F*‐value	*p*‐value	*F*‐value	*p*‐value	*F*‐value	*p*‐value
A‐Time	0.2180	.6517	0.0561	.8181	0.0904	.7704
B‐Temperature	2.17	.1750	6.14	.0351*	3.55	.0921
C‐Power	3.60	.0504*	1.80	.2121	4.32	.0574*
AB	4.25	.0693	1.38	.2699	2.15	.1765
AC	5.04	.0515*	3.22	.1063	5.73	.0404*
BC	5.46	.0443*	4.17	.0515*	4.34	.0668
A^2^	0.0005	.9826	0.0174	.8979	0.0156	.9033
B^2^	7.67	.0218*	15.24	.0036*	9.07	.0147*
C^2^	4.38	.0659	0.0174	.8979	1.69	.2256

* *p* < .05.

**FIGURE 2:**
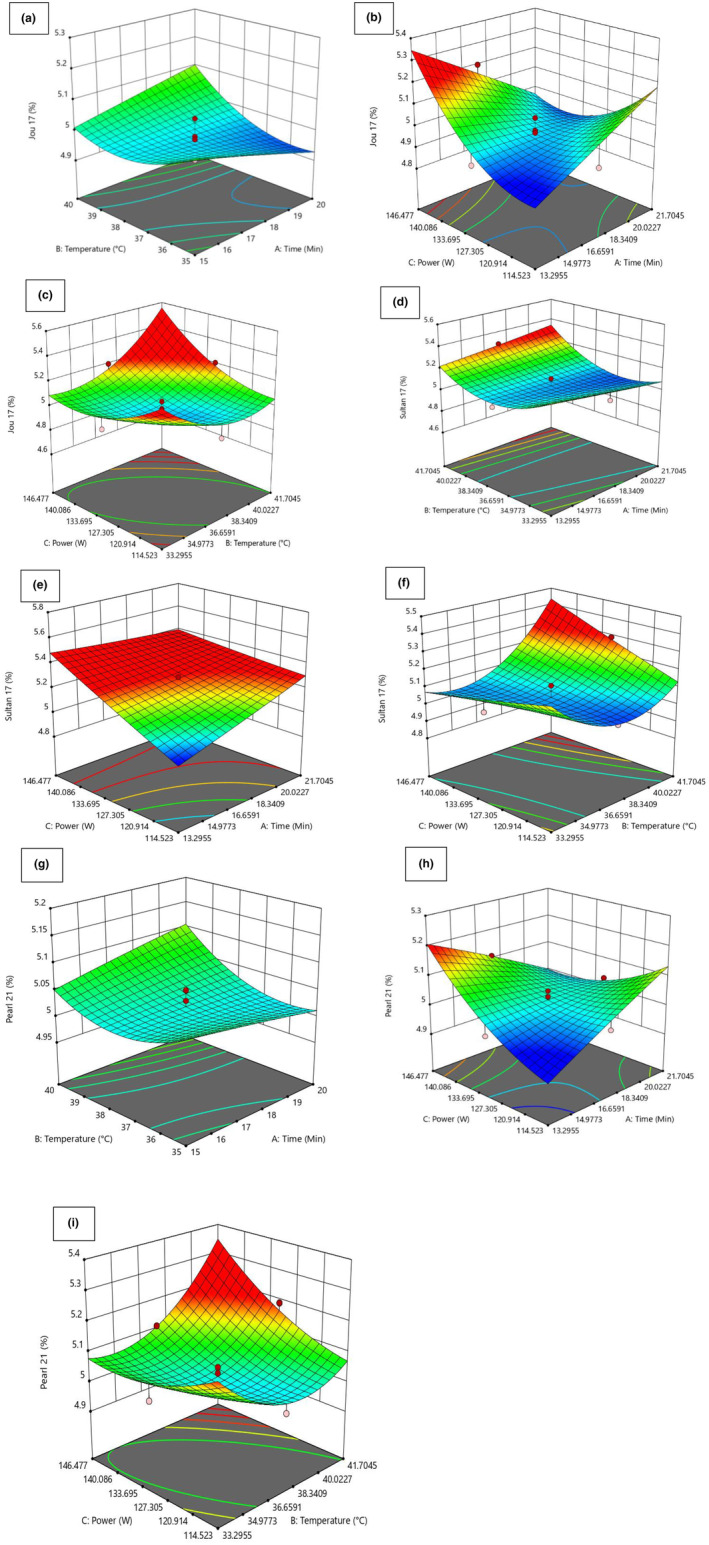
3D contour plot of optimized SDF (β‐glucan) yield from selected varieties (Jou‐17, Sultan17, Pearl‐21) with various responses. (a) Yield of Jou‐17 with time and temperature. (b) Yield of Jou‐17 with time and power. (c) Yield of Jou‐17 with temperature and power. (d) Yield of Sultan‐17 with time and temperature. (e) Yield of Sultan‐17 with time and power. (f) Yield of Sultan‐17 with temperature and power. (g) Yield of Pearl‐21 with time and temperature. (h) Yield of Pearl‐21 with time and power. (i) Yield of Pearl‐21 with temperature and power.

In the current research, significant insights were assembled from Table 3, revealing that the linear coefficients (C), cross‐product coefficients (AC and BC), and quadratic term coefficients (B^2^) showed significance; (*p* < .05). Conversely, the other coefficients did not reach the threshold of significance (*p* > .05). This underscored the importance of C, AC, BC, and B^2^ as pivotal factors in the extraction process of SDF (β‐glucan). To optimize the yield of SDF (β‐glucan), Design‐Expert software was used to generate 3D graphical representations of the regression Equation (2). These visualizations showcased the influence of extraction temperature, extraction time, and power on the extraction yield of SDF (β‐glucan, as depicted in the accompanying Figure 2).

In these 3D response surface plots, it was apparent that when power (C) remained fixed at zero, extraction time (A) and extraction temperature (B) exhibited a reciprocal interaction, signifying their quadratic effects on the extraction yield (Figure 2a). Furthermore, when the extraction temperature (B) kept stable, the increase in the SDF (β‐glucan) yield was noted with high power (C) with no discernible impact of extraction time (A) (Figure 2b). Figure 2c illustrates that the extraction temperature (B) and power (C) demonstrated quadratic effects on the yield of SDF (β‐glucan) when extraction time (A) was held at a constant zero level that led to an increase in yield with higher levels of power (C).

Furthermore, significant insights were assembled from Table 3, revealing that the linear coefficients (B), cross‐product coefficients (BC), and quadratic term coefficients (B^2^) showed significance; (*p* < .05). Conversely, the other coefficients did not reach the threshold of significance (*p* > .05). This underscored the importance of B, BC, and B^2^ as pivotal factors in the extraction process of SDF (β‐glucan) from Sultan‐17. To optimize the yield of SDF (β‐glucan), Design‐Expert software was used to generate 3D graphical representations of the regression Equation (1). These visualizations showcased the influence of extraction temperature, extraction time, and power on the extraction yield of SDF (β‐glucan, as depicted in the accompanying Figure 2d–f).

In the context of these 3D response surface plots, it became clear that when the power level (C) was kept at zero, the extraction time (A) and extraction temperature (B) displayed a reciprocal interaction within the initial range, but as the values extended beyond that range, they demonstrated a direct interaction, indicated the influence on the extraction yield followed a quadratic pattern (Figure 2d). Furthermore, when the extraction temperature (B) remained at zero level, the yield increased as time (A) and power (C) were raised (Figure 2e). Figure 2f illustrates that the extraction temperature (B) and power (C) demonstrated quadratic effects on the yield of SDF (β‐glucan) when extraction time (A) was held at a constant zero level that led to an increase in yield with higher levels of power (C).

Furthermore, significant insights were assembled from Table 3, revealing that the linear coefficients (C), cross‐product coefficients (AC), and quadratic term coefficients (B^2^) showed significance; (*p* < .05). Conversely, the other coefficients did not reach the threshold of significance (*p* > .05). This underscored the importance of B, BC, and B^2^ as pivotal factors in the extraction process of SDF (β‐glucan) from Pearl‐21. To optimize the yield of SDF (β‐glucan), Design‐Expert software was used to generate 3D graphical representations of the regression Equation (1). These visualizations showcased the influence of extraction temperature, extraction time, and power on the extraction yield of SDF (β‐glucan, as depicted in the accompanying Figure 2g–i).

In the context of these 3D response surface plots, it became clear that when the power level (C) was kept at zero, the uniform extraction time (A) behavior with increasing extraction temperature (B) displayed an interaction, indicating the influence on the extraction yield followed a quadratic pattern (Figure 2g). Furthermore, when the extraction temperature (B) remained at zero level, the yield increased as time (A) and power (C) were raised (Figure 2h). Figure 2i illustrates that the extraction temperature (B) and power (C) demonstrated quadratic effects on the yield of SDF (β‐glucan) when extraction time (A) was held at a constant zero level that led to an increase in yield with higher levels of temperature (B) power (C).

Based on the findings presented in Figure 2, it was concluded that the optimal extraction conditions for SDF (β‐glucan) from the dehulled barley varieties (Jou‐17, Sultan‐17, and Pearl‐21) included an extraction temperature of 41.70°C, an extraction time of 17.5 min, and a power of 130.5 Watts. Among the three extraction parameters investigated, power and temperature emerged as the most influential factors in affecting the extraction yield of SDF (β‐glucan). This conclusion was verified by the significance of the regression coefficients outlined in the quadratic polynomial model (Tables 2 and 3) and the slopes observed in the 3D response surface plots (Figure 2).

### 5.2 Fourier transform infrared spectroscopy (FTIR)

**FIGURE 3:**
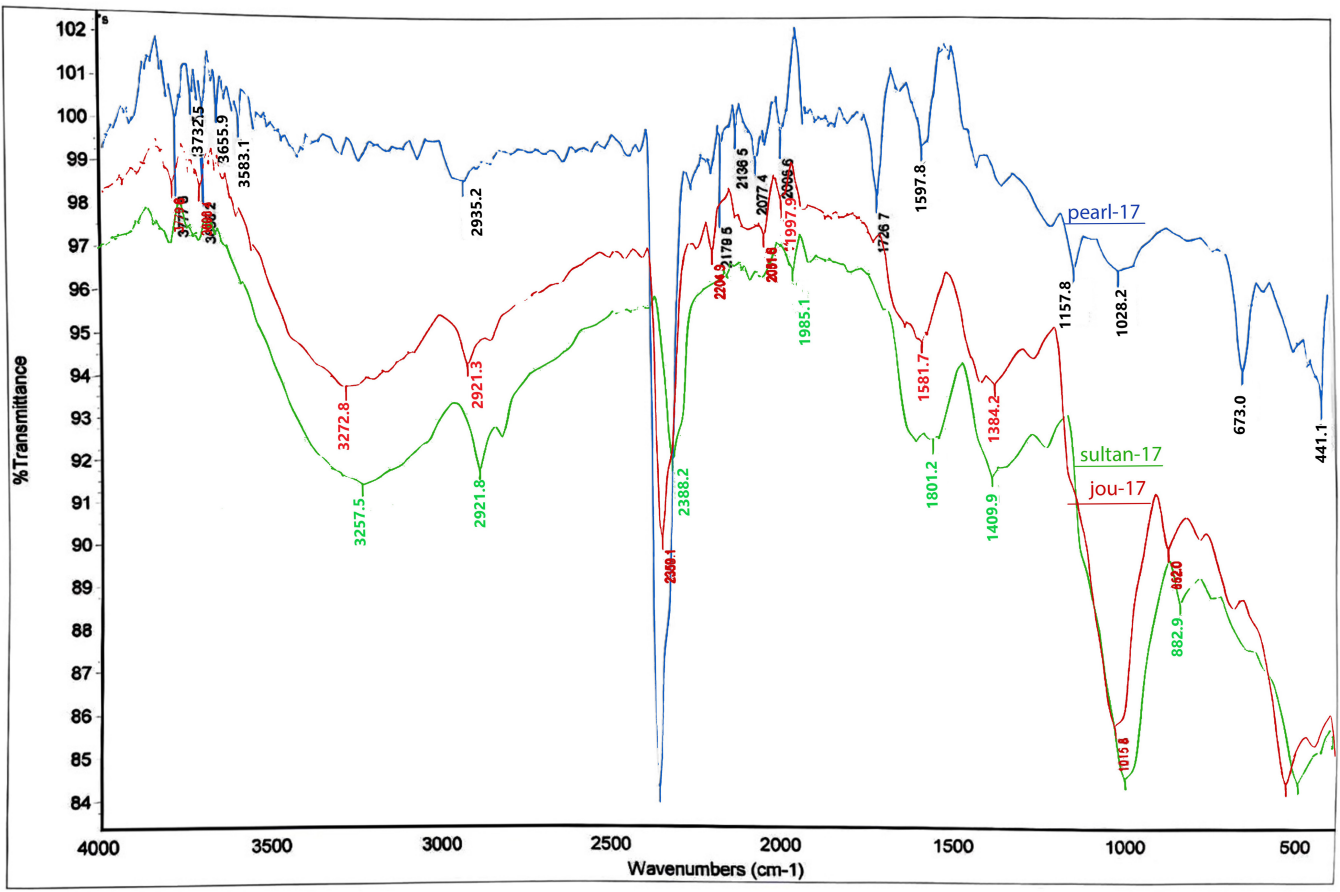
FTIR patterns of SDF (β‐glucan) extracted from selected barley varieties (Jou‐17, Sultan‐17, and Pearl‐21).

Figure 3 describes the Fourier transform infrared spectroscopy (FT‐IR) analysis of SDF of (β‐glucan) from the barley varieties (Jou‐17, Sulatn‐17, and Pearl‐21). SDF (β‐glucan) was composed of polysaccharides and exhibited distinct absorption peaks within the range of 4000–400 cm^−1^, as reported by Ahmad et al. (2010). The FT‐IR analysis revealed that all SDF (β‐glucan) from Jou‐17 and Sultan‐17 samples exhibited absorption peaks at specific wavenumbers: approximately 3227 and 3257 cm^−1^, corresponding to O‐H stretching; 2921 cm^−1^, indicative of CH and CH_2_ stretching, in accordance with the findings of Limberger‐Bayer et al. (2014). Notably, the presence of linked β (1 → 3) and β (1 → 4) structures, forming the backbone of barley β‐glucan from ou‐17 and Sultan‐17, was evident from the absorption peak at 862 cm^−1^, as described by Ahmad et al. (2010).

Furthermore, the FT‐IR spectra of SDF (β‐glucan) of all selected barley varieties showed peaks around 1681, 1581, and 1408 cm^−1^, likely associated with NH and CN group stretching, suggesting the presence of proteins, consistent with the observations of Abdel‐Haleem et al. (2020); Ahmad et al. (2010). The most notable distinction observed in the FT‐IR analysis of SDF (β‐glucan) was with greater intensity and broader width of the absorption peaks for the –OH and –CH groups and CH_2_ stretching in the SDF (β‐glucan) from the Jou‐17 cultivar, as shown in Figure 5. Additionally, the SDF (β‐glucan) exhibited a unique absorption peak at 2359 cm^−1^, from all selected barley varieties associated with bicarbonate groups (Liu, 2019).

### 5.3 Monosaccharide composition of SDF (β‐glucan)

**FIGURE 4:**
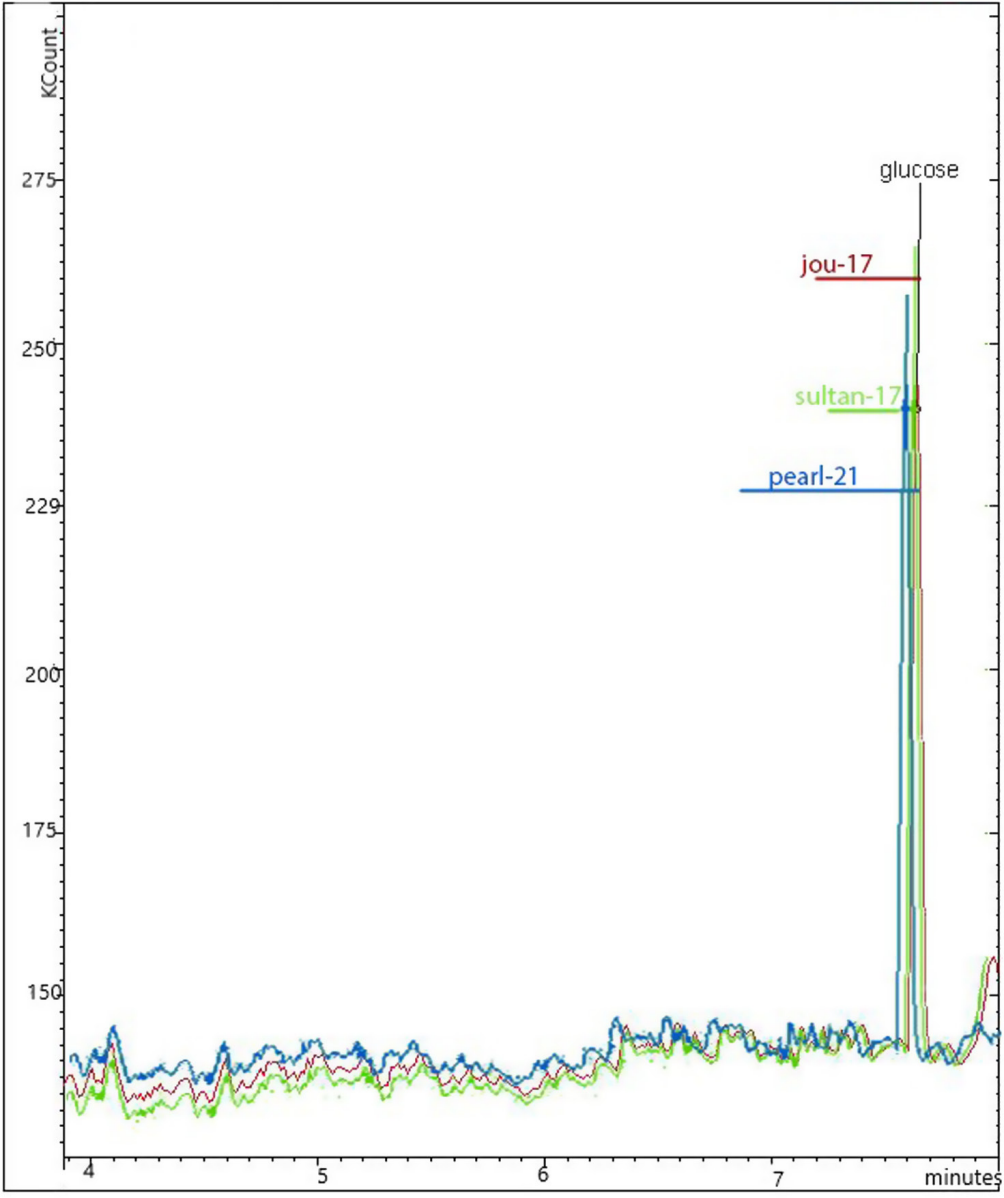
Monosaccharide composition of SDF (β‐glucan) extracted from selected barley varieties (Jou‐17, Sultan‐17, and Pearl‐21).

The monosaccharide composition profile of SDF (β‐glucan) is presented in Figure 4. The findings indicate that all selected barley varieties (Jou‐17, Sultan‐17, and Pearl‐21) of SDF (β‐glucan) are primarily composed of glucose and showed a similar peak ranging from 230 to 270 × 10^3^ count. Couture et al. (2022) confirmed the fiber composition that somewhat aligned with current study findings. Joye (2020) also described the similar monosaccharide composition of β‐glucan extracted from whole wheat.

### 5.4 Differential scanning calorimetry

**FIGURE 5:**
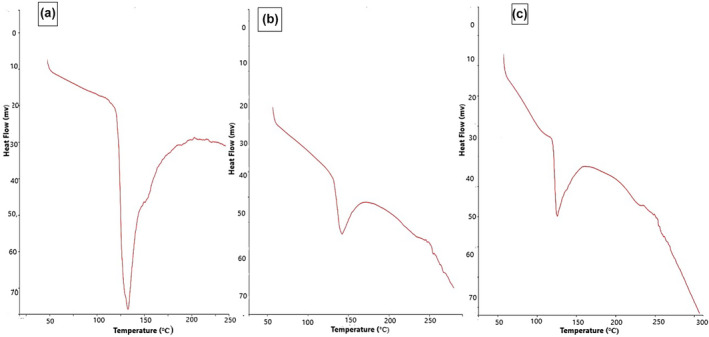
DSC graph of β‐glucan extracted from selected barley varieties (a) Jou‐17, (b) Sultan‐17, and (c) Pearl‐21.

Differential scanning calorimetry (DSC) was employed to investigate the thermal transitions that occur during the heating process under a nitrogen atmosphere (Figure 5). The thermograms depicted in Figure 5 represent the DSC profile of SDF (β‐glucan) derived from selected barley varieties (Jou‐17, Sultan‐17, and pearl‐21). An endothermic response of SDF of Jou‐17 (Figure 5a) was detected at 59°C, whereas in the case of SDF of Sultan‐17 (Figure 5b) and Pearl‐21 (Figure 5c), the response was observed at 50°C which is attributed to the evaporation of water (Kagimura et al., 2015). Notably, a substantial endothermic phenomenon in all varieties was also observed at a higher temperature, specifically at 130°C (Abdel‐Haleem et al., 2020).

### 5.5 Thermogravimetric analysis

**FIGURE 6:**
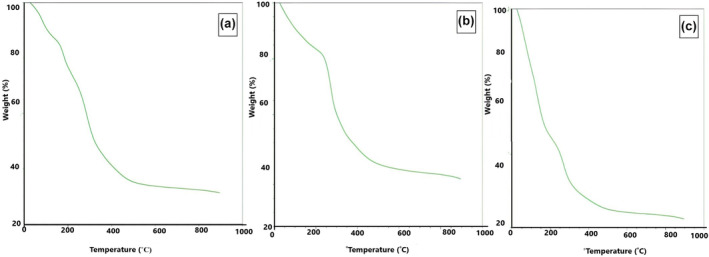
TGA graph of β‐glucan extracted from selected barley varieties (a) Jou‐17, (b) Sultan‐17, and (c) Pearl‐21.

During the initial phase of the TGA analysis (Figure 6), a minor reduction in weight was detected around 170°C in all selected barley varieties. This weight loss was attributed to the evaporation and subsequent removal of moisture from the SDF (β‐glucan) of selected barley varieties sample. Subsequently, a significant weight loss of approximately 30% in the Jou‐17 variety (Figure 6a) was observed within the temperature range of 220–450°C whereas about 45% weight loss was observed in the other two varieties (Sultan‐17 and Pearl‐21) (Figure 6b,c). This particular interval is indicative of the thermal decomposition and breakdown of the β‐glucan polymer structure and carbonization that indicated the thermal stability of the SDF (β‐glucan). The disruption of the hydrogen bonds and other non‐covalent interactions that stabilize the polymer chains can lead to the dissolution of chemical bonds and fragmentation of the polymer chains (Zhao et al., 2022). This pattern decreases the molecular weight of the β‐glucan and alters its physical and chemical properties. Further, the onset of carbonization may mark the start of combustion of the β‐glucan sample, leading to a noticeable decrease in weight as observed in the data (Labafzadeh, 2015). Following this notable weight loss in the sample, marginal fluctuations in weight were observed up to 800°C in the sample. A steady weight loss at this temperature indicated a change in the structure of SDF (β‐glucan) (Zhao et al., 2022).

### 5.6 Proximate analysis and functional properties

Proximate analysis is an essential analytical method used to determine the basic composition of a food sample. Proximate analysis and functional properties of SDF (β‐glucan) of the selected barley varieties (Jou‐17, Sultan‐17, and Pearl‐21) are presented in Table 4. The results of the SDF (β‐glucan) showed differences in the constituents, including the values of ash, moisture, fat, crude protein, crude fat, and nitrogen‐free extract being 0.79%, 13.40%, 0.3%, 7.42%, 10.10%, and 77.98% for Jou‐17 barley cultivar. Meanwhile, the results of the SDF (β‐glucan) extracted from the Sultan‐17 variety showed differences in the constituents, including the values of ash, moisture, fat, crude protein, crude fat, and nitrogen‐free extract being 2.75%, 13.48%, 1.22%, 8.84%, 10.02%, and 73.68% respectively. However, Kanwal et al. (2023) reported the ash percentage in barley‐17 and Sultan‐17 as 1.59% and 1.8% respectively. Furthermore, ash, moisture, fat, crude protein, crude fat, and nitrogen‐free extract content were recorded in the Pearl‐21 variety as 0.68%, 13.51%, 0.32%, 7.02%, 10.08%, and 78.37% respectively. The findings of the current study were consistent with the findings of the reported study (Nogala‐Kałucka et al., 2020) on proximate analysis of wheat, barley, and oat varieties.

**TABLE 4:** The proximate analysis and functional properties of SDF (β‐glucan) extracted from dehulled barley grains (Jou‐17, Sultan‐17, and Pearl‐21).

SDF (β‐Glucan) of selected barley varieties	Proximate analysis						Functional properties			
	Ash (%)	Moisture (%)	Crude fat (%)	Crude protein (%)	Crude fiber (%)	Nitrogen‐free extract (%)	Water holding capacity (WHC) g/g	Oil holding capacity (OHC) g/g	Foaming capacity (FC) %	Foaming stability (FS) %
Jou‐17	0.79 ± 0.015^b^	13.40 ± 0.07^a^	0.31 ± 0.0081^b^	7.42 ± 1.38^b^	10.10 ± 0.031^a^	77.98 ± 1.99^b^	13.47 ± 1.29^b^	6.83 ± 0.84^b^	121.41 ± 3.79^c^	96.26 ± 2.15^a^
Sultan‐17	2.75 ± 0.01^a^	13.48 ± 0.02^b^	1.22 ± 0.001^a^	8.84 ± 2.13^a^	10.02 ± 0.091^c^	73.68 ± 2.18^c^	13.29 ± 1.11^c^	6.19 ± 0.43^c^	123.04 ± 4.12^b^	93.49 ± 3.29^c^
Pearl‐21	0.68 ± 0.001^c^	13.51 ± 0.01^b^	0.32 ± 0.009^b^	7.02 ± 1.88^c^	10.08 ± 0.005^b^	78.37 ± 2.30^a^	14.07 ± 1.37^a^	6.99 ± 0.16^a^	126.17 ± 4.77^a^	93.71 ± 3.02^b^

*Note*: Different notations (a–c) show significant differences in the proximate analysis and functional properties at a 95% probability level.

The WHC and OHC of ultra‐sonicated extracted SDF (β‐glucan) in the “Jou‐17” variety of barley were determined to be 13.47 and 6.83 g/g, respectively. The foaming capacity (FC) and foaming stability (FS) of SDF (β‐glucan) in the “Jou‐17” were measured at 121.41 and 96.26%, respectively. The other two varieties (Sultan‐17 and Pearl‐21) exhibited WHC and OHC of ultra‐sonicated extracted SDF (β‐glucan) as 13.29 and 14.07 g/g, and 6.19 and 6.99 g/g, respectively. The foaming capacity (FC) and foaming stability (FS) of SDF (β‐glucan) in the “Sultan‐17” were measured at 123.04% and 93.49% respectively. Similarly, the foaming capacity (FC) and foaming stability (FS) of SDF (β‐glucan) in the “Pearl‐21” were measured at 126.17% and 93.71%, respectively. The previous literature (Abdel‐Haleem et al., 2020) supported the current study findings in terms of FC and FS by reporting almost similar functional values of SDF (β‐glucan) of different barley varieties ranging from 112% to 131% (FC) and 97% to 100% (FS). Similarly, the WHC and OHC reported by Zhang et al. (2022) were in line with 10 and 5 g/g which were quite close to the current study findings. The intense energy from ultrasound waves may cause disruptions or changes in the polymer structure of β‐glucan, affecting WHC and OHC (Martinez‐Solano et al., 2021).

### 5.7 Principal component analysis (PCA)

**FIGURE 7:**
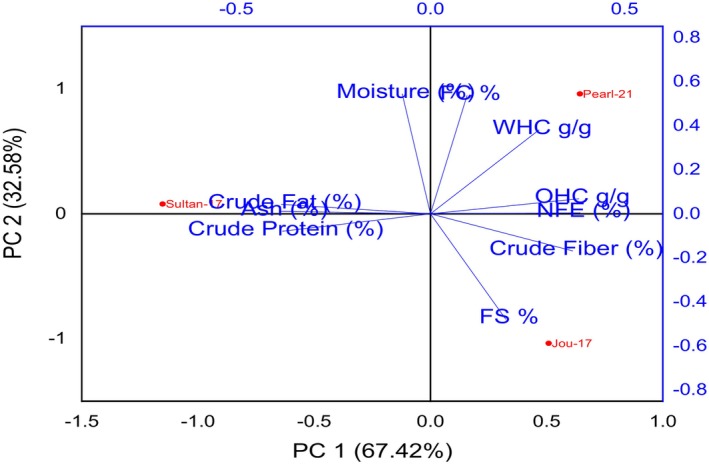
Principal component analysis (PCA) of studied variables observed in SDF (β‐glucan) in selected barley varieties.

In this study, the principal component analysis (PCA) technique was applied to assess the relationship between proximate analysis and functional properties in SDF (β‐glucan) derived from specific barley varieties, namely Jou‐17, Sultan‐17, and Pearl‐21. Figure 7 visually depicts a significant relationship among these variables. The analysis involved extracting eigenvalues from the original data, with the scree plot clearly illustrating a decreasing trend in the graph line for all the factors under investigation.

Notably, the cumulative variance explained by the first two principal components (PC) out of the 10‐dimensional variables was 100%. PC1 accounted for 67.42% of the variance, while PC2 explained 32.58% of the variation. The results indicated that the Pearl‐21 variety exhibited a positive and strong correlation among studied variables, including moisture (%), FC (%), WHC, OHC, and NFE (%). In contrast, the Jou‐17 variety highlighted its association with FS (%) and Crude fiber (%), while the Sultan‐17 variety displayed a weak and negative correlation with crude fat (%), ash (%), and crude protein (%). Ramdath et al. (2020) described the PCA among different lentils (cereal and grains) to examine the variance within the proximate analysis.

## 6 CONCLUSION

In a nutshell, optimizing the extraction of SDF (β‐glucan) from the selected barley cultivars using green technology and its favorable properties opens up promising paths for future endeavors. The focus can shift toward further exploring the functional and nutritional benefits of SDF (β‐glucan) in food products and dietary applications. Additionally, research delved into scaling up of production processes and investigating potential health‐related benefits, ultimately contributing to the advancement of sustainable and health‐conscious practices in the food industry.

## AUTHOR CONTRIBUTIONS

**Nomeena Anis:** Conceptualization (equal); data curation (equal); formal analysis (equal); funding acquisition (equal); methodology (equal); project administration (equal); writing – original draft (equal); writing – review and editing (equal). **Zaheer Ahmed:** Conceptualization (equal); investigation (equal); methodology (equal); resources (equal); supervision (equal); writing – review and editing (equal). **Nauman Khalid:** Methodology (equal); supervision (equal); writing – review and editing (equal).

## CONFLICT OF INTEREST STATEMENT

There is no conflict of interest among the authors of this study.

## Data Availability

Data will be provided on request.
